# Propofol inhibits invasion and growth of ovarian cancer cells via regulating miR-9/NF-κB signal

**DOI:** 10.1590/1414-431X20165717

**Published:** 2016-12-12

**Authors:** X. Huang, Y. Teng, H. Yang, J. Ma

**Affiliations:** 1 Department of Gynecology, the People’s Hospital of Laiwu, Jinan, China; 2 Department of Oncology, the People’s Hospital of Rizhao, Rizhao, China; 3 Department of Gynecology and Obstetrics, the First People’s Hospital of Jinan, Jinan, China; 4 Department of Clinical Laboratory, the People’s Hospital of Weifang, Weifang, China

**Keywords:** Propofol, Ovarian cancer, miR-9, NF-κB, Apoptosis, Invasion

## Abstract

Propofol is one of the most commonly used intravenous anesthetic agents during cancer resection surgery. A previous study has found that propofol can inhibit invasion and induce apoptosis of ovarian cancer cells. However, the underlying mechanisms are not known. miR-9 has been reported to be little expressed in ovarian cancer cells, which has been related to a poor prognosis in patients with ovarian cancer. Studies have also demonstrated that propofol could induce microRNAs expression and suppress NF-κB activation in some situations. In the present study, we assessed whether propofol inhibits invasion and induces apoptosis of ovarian cancer cells by miR-9/NF-κB signaling. Ovarian cancer ES-2 cells were transfected with anti-miR-9 or p65 cDNA or p65 siRNA for 24 h, after which the cells were treated with different concentrations of propofol (1, 5, and 10 μg/mL) for 24 h. Cell growth and apoptosis were detected using MTT assay and flow cytometry analysis. Cell migration and invasion were detected using Transwell and Wound-healing assay. Western blot and electrophoretic mobility shift assay were used to detect different protein expression and NF-κB activity. Propofol inhibited cell growth and invasion, and induced cell apoptosis in a dose-dependent manner, which was accompanied by miR-9 activation and NF-κB inactivation. Knockdown of miR-9 abrogated propofol-induced NF-κB activation and MMP-9 expression, reversed propofol-induced cell death and invasion of ES-2 cells. Knockdown of p65 inhibited NF-κB activation rescued the miR-9-induced down-regulation of MMP-9. In addition, overexpression of p65 by p65 cDNA transfection increased propofol-induced NF-κB activation and reversed propofol-induced down-regulation of MMP-9. Propofol upregulates miR-9 expression and inhibits NF-κB activation and its downstream MMP-9 expression, leading to the inhibition of cell growth and invasion of ES-2 cells.

## Introduction

Malignant tumor metastasis consists of a series of biological occurrences, of which an important one is the presence of circulating tumor cells (CTCs) that are released from the primary tumor into the bloodstream (1). The presence of CTCs in the blood represents a poor clinical outcome in a variety of carcinomas, including ovarian cancer (2), due to the seeding of distant organs and subsequent overgrowth in the new microenvironment (3).

Numerous studies have recently found that tumor cells intravasate, rapidly transit through the circulation, and arrest in the vasculature of a secondary organ during operation, generally taking a few minutes (4–6). In addition, platelets form aggregates around CTCs or arrest tumor cells during this period. It was recently reported that 7–48 h after tail-vein injection of tumor cells, monocytes/macrophages are also recruited to their vicinity. Extravasation typically takes place within the first 24–72 h after initial arrest. By that time, most tumor cells have exited the bloodstream and seeded into the stroma of the secondary site (7). The invasion of tumor cells in the circulation may occur very early in tumor development. However, current therapy is not altered based on CTC status. A lack of understanding of the biology of CTCs has served as a barrier to developing rational therapy tailored to these high-risk patients.

Propofol, the intravenously administered hypnotic agent, is widely used in all kinds of surgeries due to its short effect and rapid recovery. Patients receiving total intravenous anesthesia (TIVA) with propofol have been shown to experience less postoperative pain. Accumulating clinical evidence indicates that propofol TIVA for cancer surgery reduces the risk of recurrence or metastasis during the initial years of follow-up (8–11), indicating that propofol has the effect to kill cancer cells released into the circulation in the perioperative period.

Propofol functions involve various mechanisms. Some *in vitro* evidence suggested that exposure to propofol induced significant cell death in the hESC-derived neurons by regulation of microRNAs expression (12). Recently, it was found that inactivation of the NF-κB signaling by propofol abrogated gemcitabine-induced activation of NF-κB, resulting in the chemosensitization of pancreatic cancer cells to gemcitabine (13). In aggressive ovarian cancers, NF-κB and NF-κB target gene MMP-9 are activated (14,15). In addition, activation of NF-κB signaling could increase aggressiveness of ovarian cancer cells, and vice versa (16).

MicroRNAs (miRs) are small non-coding RNAs, regulating gene expression post-transcriptionally. They mediate fundamental cellular processes such as proliferation, differentiation and apoptosis and are actively involved in carcinogenesis (17). miR-9 was recently implicated in cancers. It has been reported to be little expressed in ovarian cancer (18,19). Overexpression of miR-9 could induce anti-proliferative, anti-invasive, and pro-apoptotic activity (20). miR-9 directly targeted NF-κB mRNA and suppressed expression of both p65 and p50 subunits of NF-κB in ES-2 cells (19). Down-regulation of miR-9 in ovarian cancer cells was shown to contribute to NF-κB activation (19).

In the present study, we assessed the effect of propofol on apoptosis, growth and invasion of ovarian cancer cells *in vitro*, and explored its molecular mechanisms.

## Material and Methods

### Cell line and culture

Human ovarian cancer ES-2 cell line was purchased from the Type Culture Collection of Chinese Academy of Sciences (Shanghai, China). It was grown in RPMI-1640 (Gibco, China) supplemented with 10% FBS, 100 IU/mL of penicillin and 100 μg/mL of streptomycin, and incubated at 37°C in 95% humidity chamber supplemented with 5% CO_2_.

### anti-miR-9 transfection

Both miR-9 inhibitor (anti-miR-9) and the scrambled miR-9 inhibitor (negative control) were purchased from Applied Biosystems (China) and used according to the manufacturer’s instruction. ES-2 cells (3×10^5^) were transiently transfected with anti-miR-9 (100 nM) or negative control (100 nM) with siPORT™ NeoFX™ Transfection Agent (Applied Biosystems; 10 µL in 200 µL of OPTI-MEM¯ I medium without serum) for 48 h. Cells were then harvested and analyzed.

### Plasmid transfection

Full-length human RelA cDNA was amplified by PCR from pCMV4-RelA plasmid (Addgene, China) using forward primer 5′-GGTCGGTACCATGGACGAACTGTTCCCCCT-3′ and reverse primer 5′-CCATCTCGAGTTAGGAGCTGATCTGACTCA-3′, inserted into pcDNA3.1 vector (Invitrogen, China) tagged with FLAG. P65 siRNA, MMP-9 siRNA and its control siRNA was purchased from Santa Cruz Biotechnology (China). Transient transfection of ES-2 cells with pcDNA3.1/p65 cDNA or control pcDNA3.1, P65 siRNA, MMP-9 siRNA and its control siRNA was carried out using the LipofectAmine reagent (Life Technologies, China) according to the manufacturer's instructions.

### Drug treatment

The dose of propofol used clinically varies widely but typically ranges from 1–10 μg/mL (blood concentration) with higher doses used for induction of anesthesia and lower doses used for maintenance. Thus, ES-2 cells were treated with 0, 5, and 10 μg/mL of research grade propofol (0–112 μM, Sigma-Aldrich, USA) or equal volume of dimethyl sulfoxide (DMSO, Sigma-Aldrich) as the vehicle control in 96-well plates. A stock solution (40 mg/mL) of propofol was prepared in DMSO and serial dilutions to the desired doses were prepared from the stock. Before treatment, ES-2 cells were cultured at the density of 3×10^5^ cells/dish on a 60- mm culture dishes and used 24 h later when they were 80% confluent. Cells were exposed to propofol (5 and 10 μg/mL) for 6 h. After washing, cells were then cultured in DMEM supplemented with 10% FBS and antibiotics for another 24 h. To determine the signaling pathways involved in the production of miR-9, NF-κB activity, p65 nucleus translocation and MMP-9 expression, ES-2 cells were transfected with anti-miR-9 or/and p65 cDNA, p65 siRNA, MMP-9 siRNA 24 h before propofol exposure, as described above. The cells were lysed for miR-9 analysis and western blot, EMSA, ELISA, apoptosis and invasion assay.

### Real-time PCR (qPCR)

At every experimental end point, cells were collected and washed twice with ice-cold PBS and lysed with QIAzol reagent (China) to isolate total RNA. miR-9 levels were quantified in total RNA by real-time PCR using the TaqMan miRNA qPCR Kit and primer/probe sets (Life Technologies), following the manufacturer’s instructions. Results were normalized to U6, using the relative quantitation (RQ) method.

### Electrophoretic mobility shift assay (EMSA)

Nuclear extracts were prepared according to a previous report (21). The extracts were incubated with ATP (γ-^32^P)-labeled NF-κB consensus oligonucleotides (Promega, China) in a gel-shift binding buffer for 40 min at room temperature and separated in 8% native polyacrylamide gels followed by autoradiography (22).

### Western blotting

Total protein was extracted from cultured cells in different groups as the previous report. Twenty-five micrograms of protein extracts were separated using 10% SDS-PAGE and electroblotted on a PVDF-membrane. The membranes were incubated overnight with antibodies of p65 (1:200), MMP-9 (1:200) and β-actin (1:1000) at 4°C. Then, the membranes were incubated with anti-rabbit IgG (1:5000), and exposed to X-ray film using an enhanced chemiluminescence system (ThermoFisher Scientific, China). The intensity of the bands was measured using Lab-works.

### Enzyme-linked immunosorbent assay (ELISA)

After treatment, cell culture media (supernatant) aliquots were collected for analysis. The concentrations of MMP-9 in ES-2 cell culture supernatants were determined by specific MMP-9 ELISA kits (Thermo Scientific, China). All procedures were carried out according to the manufacturer’s protocols.

### Flow cytometer for apoptosis assay

Using Annexin V-FITC apoptosis detection kit (Becton-Dickinson Biosciences, China), Annexin V-staining followed by a FACScan flow cytometer was used to detect cell apoptosis according to the manufacturer’s instructions. The CellQuest software was used to analyze the data (Becton-Dickinson).

### Cell viability assay

The viability of ES-2 cells in different groups was quantitatively assessed by MTT assay. The cells were incubated in 500 mg/mL MTT solution for 4 h. After solubilization of formazan crystals in DMSO, the absorbance of each well was determined by a spectrophotometric reader at 570 nm (Senago, China).

### Invasion assay

After transfection as described above, ES-2 cells were detached and washed twice in PBS. Cells (5×10^5^) were seeded in the upper chamber of a Transwell insert (12 μM pores) coated with Matrigel (0.7 mg/mL; Collaborative Research Inc., USA). The lower chamber was filled with 400 µL of RPMI medium. After a 24 h incubation period, the non-migrated cells in the upper chamber were gently scraped away and adherent cells present on the lower surface of the insert were stained with Hema-3 and photographed.

### Wound-healing assay

ES-2 cells were treated as described above. The cells were then grown to confluence and scratched with sterile 200 µL pipette tips. Plates were washed twice with PBS to remove detached cells and incubated in the complete growth medium without FBS. Cells migrated into the wounded area, and photographs were taken immediately (0 h) and at 24 h.

### Statistical assay

Statistical significance was assessed using the two-tailed *t*-test. P<0.05 was considered to be significant.

## Results

### Propofol induced apoptosis and inhibited viability of ES-2 cells

**Figure 1:**
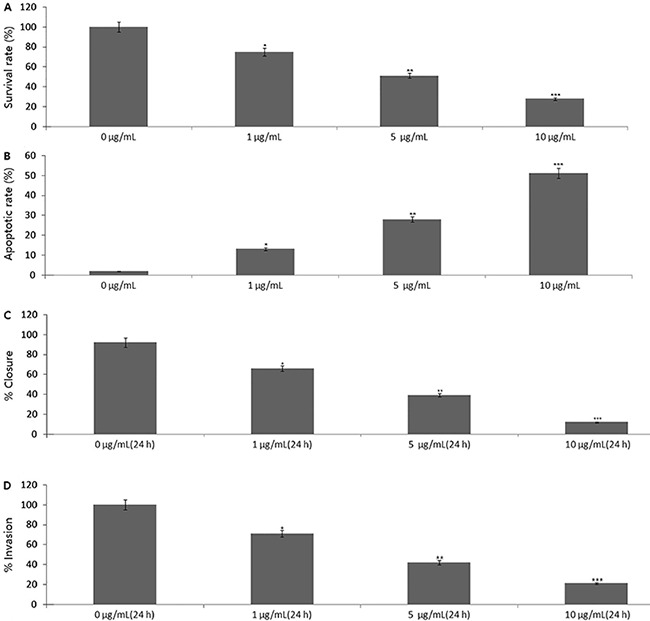
Results of ES-2 cells treated with 1, 5, and 10 μg/mL propofol for 24 h. *A*, Cell viability by MTT assay. *B*, Cell apoptosis using Annexin V-staining followed by a FACScan flow cytometer assay. *C*, Migration by wound healing migration assay. *D*, Transwell invasion assay *vs* untreated cells (0 μg/mL). *P<0.05, **P<0.01, ***P<0.001 (*t*-test).

ES-2 cells were treated with 1, 5 and 10 μg/mL propofol for 24 h. As shown in Figure 1A, after 24 h treatment, cell viability was remarkably inhibited in a dose-dependent manner. Regulation of apoptosis in ES-2 cells was analyzed using Annexin V-staining followed by a FACScan flow cytometer assay. The results showed a significant induction of apoptosis/cell death by propofol treatment. The cell apoptotic rate was 13.8±3.6, 28.4±5.1, and 49.2±4.8 after treatment of ES-2 cells with 1, 5, and 10 μg/mL propofol, respectively, for 24 h. As shown in Figure 1B, propofol induced apoptosis of ES-2 cells in a dose-dependent manner.

### Propofol inhibited migration and invasion of ES-2 cells

We first tested the effect of propofol on cell migration in ES-2 cells using a wound-healing assay (Figure 1C). Compared to untreated cells, cells treated with 1, 5, and 10 μg/mL propofol for 24 h exhibited a significantly decreased migration rate. Treatment with 10 μg/mL propofol showed the lowest migration rate among the groups.

We then examined the effect of propofol on invasion of ES-2 cells using BD Biocoat growth factor-reduced Matrigel invasion chamber assay. ES-2 cells treated with 1, 5, and 10 μg/mL propofol for 24 h showed a dose-dependent reduction in invasion ability; the number of cells that penetrated the laminin layer and passed through the bottom membrane was 71.4±7.3, 42.6±3.8, and 21.4±4.3% of the untreated controls (Figure 1D). These experiments indicate that propofol inhibited cell migration and invasion activity *in vitro*.

### Propofol activated miR-9 expression of ES-2 cells

**Figure 2:**
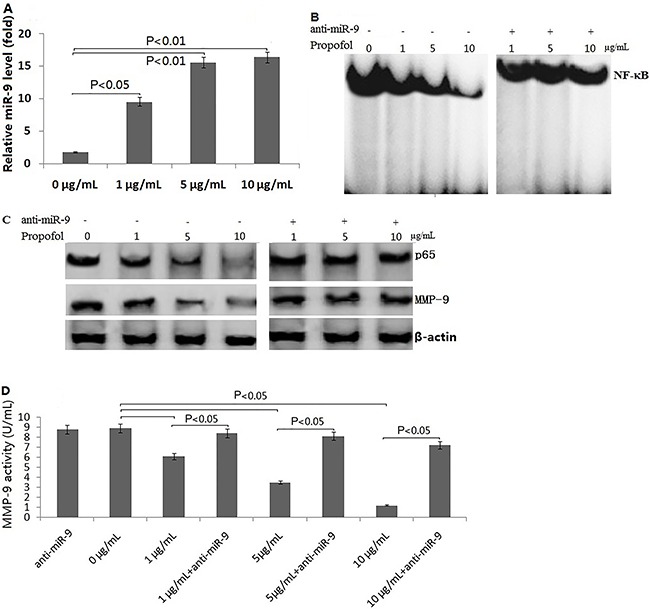
Propofol regulated miR-9-dependent NF-κB and MMP-9 expression. ES-2 cells were treated with propofol (1, 5, and 10 μg/mL) for 24 h, or for 6 h, then treated with anti-miR-9 for 24 h. *A*, miR-9 expression was detected by qRT-PCR assay. *B*, NF-κB activity was detected by EMSA. *C*, p65 and MMP-9 protein expression was detected by western blot assay. *D*, MMP-9 activity was detected by ELISA. The *t*-test was used for statistical analysis.

As shown in Figure 2A, less miR-9 expression was shown in the ES-2 cells. When treated with 1, 5, and 10 μg/mL propofol for 24 h, miR-9 expression was significantly increased in a dose-dependent manner by QRT-PCR assay.

### Propofol inhibited NF-κB activation, p65 translocation and MMP-9 expression of ES-2 cells

As shown in Figure 2B, treatment of ES-2 cells with 1, 5, and 10 μg/mL propofol for 24 h decreased NF-κB activity in a dose-dependent manner by EMSA assay. In addition, p65 and MMP-9 protein was also significantly decreased in a dose-dependent manner by western blot assay (Figure 2C). Supernatant MMP-9 activity was also decreased in a dose-dependent manner by ELISA assay (Figure 2D).

### Propofol inhibited NF-κB and MMP-9 expression through activating miR-9

We found that after anti-miR-9 transfection, followed by propofol treatment, NF-κB activity (Figure 2B), p65 and MMP-9 protein expression (Figure 2C), and MMP-9 activity (Figure 2D) were significantly increased. In the pre-experiment, we had found that scrambled miR-9 inhibitor (negative control) did not affect p65, MMP-9 and NF-κB levels, so we did not show the effect of scrambled miR-9 inhibitor in the present study.

### Propofol inhibited NF-κB-dependent MMP-9 expression

Anti-miR-9/ES-2 cells were transfected with p65 siRNA for 24 h and then treated with propofol (1, 5, and 10 μg/mL) for 24 h. The results showed that targeting p65 by p65 siRNA, transfection inhibited NF-κB activity and p65 expression, and rescued the propofol-induced down-regulation of MMP-9 protein expression and MMP-9 activity.

**Figure 3:**
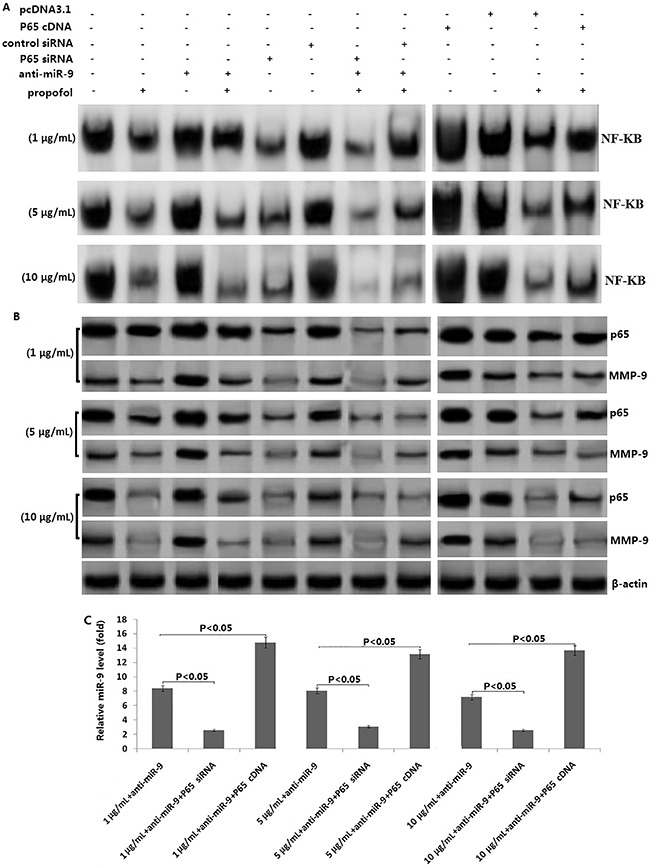
Effect of propofol on NF-κB -dependent MMP-9 expression. Anti-miR-9/ES-2 cells were transfected with p65 siRNA or P65 cDNA and its control for 24 h, then treated with propofol (1, 5, and 10 μg/mL) for 6 h. *A*, NF-κB activity was detected by EMSA in siRNA or cDNA transfected cells; *B*, p65 and MMP-9 protein expression was detected by western blot assay in siRNA or cDNA transfected cells; *C*, MMP-9 activity was detected by ELISA. The t-test was used for statistical analysis.

In addition, overexpression of p65 by p65 cDNA transfection increased propofol-induced NF-κB activity and reversed the propofol-induced down-regulation of MMP-9 protein expression and MMP-9 activity. However, the control siRNA or control pcDNA3.1 transfection did not have any effect on NF-κB activity, p65 and MMP-9 expression as well as MMP-9 activity (Figure 3).

### Apoptosis-enhancing effect of propofol was mediated by miR-9/NF-κB signal

**Figure 4:**
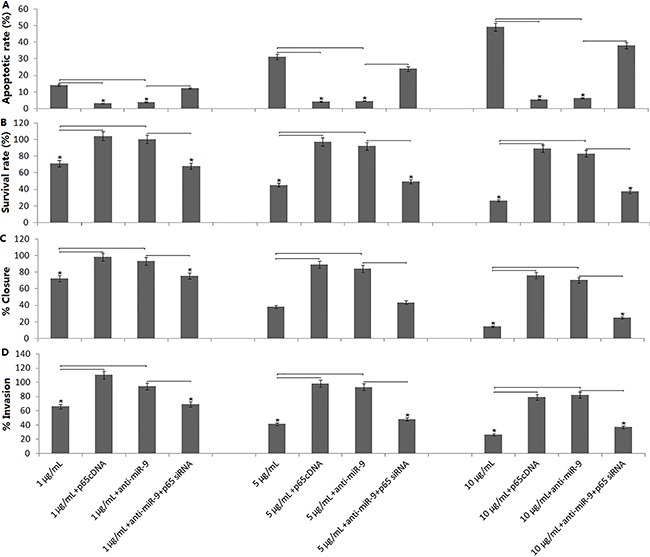
Effect of miR-9/NF-κB /MMP-9 signal on propofol-induced apoptosis and invasion. ES-2 cells were transfected with anti-miR-9 or p65 siRNA or p65 cDNA for 24 h, and then treated with 1, 5, and 10 μg/mL propofol for 6 h. *A*, Cell apoptosis was detected using Annexin V-staining followed by a FACScan flow cytometer assay. *B*, Cell viability was detected by MTT assay. *C*, Wound healing migration assay; *D*, Transwell invasion assay. ^*^P<0.05. The *t*-test was used for statistical analysis.

Our results showed that anti-miR-9 transfection inhibited propofol-induced apoptosis and increased viability of ES-2 cells. Targeting p65 by p65 siRNA transfection rescued propofol-induced apoptosis and increased viability of ES-2 cells. However, overexpression of p65 by p65 cDNA transfection reversed propofol-induced apoptosis and increased viability of ES-2 cells (Figure 4A and B).

### Invasion suppression of propofol was mediated by miR-9/NF-κB/MMP-9 signal

As shown in Figure 4C and D, targeting miR-9 by anti-miR-9 transfection reversed propofol-induced inhibition of migration and invasion in the ES-2 cells. However, targeting p65 or MMP-9 by p65 siRNA or MMP-9 siRNA transfection rescued propofol-induced inhibition of migration and invasion in the ES-2 cells. Overexpression of p65 by p65 cDNA transfection reversed propofol-induced inhibition of migration and invasion in the ES-2 cells.

## Discussion

In the present study, we assessed the roles of propofol on human ovarian cancer ES-2 cells and explored its mechanisms. We found that: 1) exposure to propofol inhibited viability and induced significant cell apoptosis in the ES-2 cells; 2) exposure to propofol inhibited migration and invasion in the ES-2 cells; 3) targeting miR-9 or overexpression of p65 by p65 cDNA transfection significantly attenuated propofol-induced cell apoptosis and increased invasion and viability of ES-2 cells. While targeting p65 or MMP-9 by siRNA, transfection rescued propofol-induced inhibition of migration and invasion of ES-2 cells; 4) propofol activated miR-9 and inhibited miR-9-dependent NF-κB activation and MMP-9 expression.

MicroRNAs (miRNAs) are small noncoding RNAs that regulate gene expression at the post-transcriptional level by either degradation or translational repression of a target mRNA (17). The role of miR-9 in cancer biology is not well understood. Endogenous miR-9 levels are lower in breast cancer (23), gastric carcinoma (21), clear cell renal cell carcinoma (24) and ovarian cancer (18,19). Low levels of miR-9 is correlated with tumor growth, metastasis and hence poor prognosis of ovarian cancer (18,19). On the other hand, patients with higher levels of miR-9 had better chemotherapy response and longer progression-free survival (25). It also impeded DNA damage repair in ovarian cancer (25), suggesting that activation of miR-9 would be an effective method for the treatment of ovarian cancer.

Propofol is an intravenous sedative-hypnotic agent administered to induce and maintain anesthesia. It has been reported to have anticancer properties including direct and indirect suppression of the viability and proliferation of cancer cells by promoting apoptosis in some cancer cell lines (26–28). In the present study, we found that exposure of ES-2 cells to 1, 5 and 10 μM propofol for 24 h was sufficient to induce cell death. In addition, the migration and invasive ability of ES-2 cells was inhibited by propofol stimulation in a dose-dependent manner.

The mechanisms by which propofol induces apoptosis and inhibits invasion of cancer cells are not well understood. In our study, we found that miR-9 is less expressed in human ES-2 cells, and its expression is significantly increased by propofol stimulation in a dose-dependent manner. We further demonstrated that targeting miR-9 inhibited propofol-induced cell death and invasion of ES-2 cells, suggesting that miR-9 plays crucial roles in propofol-induced anti-tumor effect. Although miR-9 was related to propofol-induced anti-tumor effect, its gene signal pathway in propofol-induced toxicity has yet to be examined.

miR-9 is a well-established, negative regulator of NF-κB (29–31). It has been shown that overexpression of miR-9 inhibited NF-κB activity, and downregulation of miR-9 increased NF-κB activity (30). NF-κB p65 (p65) has been described as an important therapeutic target in cancer, and it is also a target of propofol (32). In the present study, we found that propofol induced miR-9 expression and inhibited NF-κB activity and p65 expression. To further investigate whether the enhanced cell growth inhibition and apoptosis as well as decreased invasion by propofol was mediated through the miR-9/NF-κB pathway, we conducted p65 cDNA, p65 siRNA and anti-miR-9 transfection studies. We found that p65 cDNA transfection in the propofol treated ES-2 cells increased the NF-κB activity, and inhibited propofol-induced anti-tumor effect. We also found that targeting miR-9 by anti-miR-9 transfection rescued NF-κB activity and p65 expression and inhibited propofol-induced cell death and invasion of ES-2 cells. However, p65 siRNA transfection reversed the effect of anti-miR-9 in the ES-2 cells. Therefore, our results clearly show that propofol inhibits cell invasion and cell viability, as well as induces apoptosis through miR-9/NF-κB pathway.

MMP-9 has been found to be directly associated with metastatic processes. NF-κB has been reported to regulate MMP-9 expression in ovarian cancer cells (14,15). Indeed, in the present study, we found that propofol inhibited NF-κB activity and concomitantly inhibited the expression of MMP-9. Anti-miR-9 or p65 cDNA transfection rescued MMP-9 expression and activity. In addition, targeting p65 inhibited MMP-9 expression and activity. We, therefore, concluded that propofol inhibited SE-2 cell migration and invasion by activation miR-9 and inactivation of NF-κB -dependent-MMP-9.

In our study, we found that propofol upregulated miR-9 expression in ovarian cancer ES-2 cells, by which it inhibited NF-κB activation and its downstream MMP-9 expression, leading to the inhibition of cell growth and invasion of ES-2 cells.

## References

[1] Steeg PS (2006). Tumor metastasis: mechanistic insights and clinical
challenges. Nat Med.

[2] Cui L, Kwong J, Wang CC (2015). Prognostic value of circulating tumor cells and disseminated tumor
cells in patients with ovarian cancer: a systematic review and
meta-analysis. J Ovarian Res.

[3] Paterlini-Brechot P, Benali NL (2007). Circulating tumor cells (CTC) detection: clinical impact and future
directions. Cancer Lett.

[4] Rolle A, Gunzel R, Pachmann U, Willen B, Hoffken K, Pachmann K (2005). Increase in number of circulating disseminated epithelial cells after
surgery for non-small cell lung cancer monitored by Maintrac(R) is a predictor for
relapse: A preliminary report. World J Surg Oncol.

[B05] Gupta GP, Nguyen DX, Chiang AC, Bos PD, Kim JY, Nadal C (2007). Mediators of vascular remodelling co-opted for sequential steps in
lung metastasis. Nature.

[6] Bernards R, Weinberg RA (2002). A progression puzzle. Nature.

[7] Husemann Y, Geigl JB, Schubert F, Musiani P, Meyer M, Burghart E (2008). Systemic spread is an early step in breast cancer. Cancer Cell.

[8] Labelle M, Hynes RO (2012). The initial hours of metastasis: the importance of cooperative
host-tumor cell interactions during hematogenous dissemination. Cancer Discov.

[B09] Wigmore TJ, Mohammed K, Jhanji S (2016). Long-term survival for patients undergoing volatile versus
*iv* anesthesia for cancer surgery: a retrospective
analysis. Anesthesiology.

[B10] Exadaktylos AK, Buggy DJ, Moriarty DC, Mascha E, Sessler DI (2006). Can anesthetic technique for primary breast cancer surgery affect
recurrence or metastasis?. Anesthesiology.

[11] Christopherson R, James KE, Tableman M, Marshall P, Johnson FE (2008). Long-term survival after colon cancer surgery: a variation associated
with choice of anesthesia. Anesth Analg.

[12] Biki B, Mascha E, Moriarty DC, Fitzpatrick JM, Sessler DI, Buggy DJ (2008). Anesthetic technique for radical prostatectomy surgery affects cancer
recurrence: a retrospective analysis. Anesthesiology.

[13] Twaroski DM, Yan Y, Olson JM, Bosnjak ZJ, Bai X (2014). Down-regulation of microRNA-21 is involved in the propofol-induced
neurotoxicity observed in human stem cell-derived neurons. Anesthesiology.

[14] Du QH, Xu YB, Zhang MY, Yun P, He CY (2013). Propofol induces apoptosis and increases gemcitabine sensitivity in
pancreatic cancer cells *in vitro* by inhibition of nuclear
factor-kappaB activity. World J Gastroenterol.

[15] Oh JH, Kim JH, Ahn HJ, Yoon JH, Yoo SC, Choi DS (2009). Syndecan-1 enhances the endometrial cancer invasion by modulating
matrix metalloproteinase-9 expression through nuclear factor
kappaB. Gynecol Oncol.

[16] Annunziata CM, Stavnes HT, Kleinberg L, Berner A, Hernandez LF, Birrer MJ (2010). Nuclear factor kappaB transcription factors are coexpressed and convey
a poor outcome in ovarian cancer. Cancer.

[17] Hernandez L, Hsu SC, Davidson B, Birrer MJ, Kohn EC, Annunziata CM (2010). Activation of NF-kappaB signaling by inhibitor of NF-kappaB kinase
beta increases aggressiveness of ovarian cancer. Cancer Res.

[18] Schickel R, Boyerinas B, Park SM, Peter ME (2008). MicroRNAs: key players in the immune system, differentiation,
tumorigenesis and cell death. Oncogene.

[19] Laios A, O'Toole S, Flavin R, Martin C, Kelly L, Ring M (2008). Potential role of miR-9 and miR-223 in recurrent ovarian
cancer. Mol Cancer.

[20] Guo LM, Pu Y, Han Z, Liu T, Li YX, Liu M (2009). MicroRNA-9 inhibits ovarian cancer cell growth through regulation of
NF-kappaB1. FEBS J.

[21] Selcuklu SD, Donoghue MT, Rehmet K, de Souza GM, Fort A, Kovvuru P (2012). MicroRNA-9 inhibition of cell proliferation and identification of
novel miR-9 targets by transcriptome profiling in breast cancer
cells. J Biol Chem.

[22] Lee JH, Koo TH, Yoon H, Jung HS, Jin HZ, Lee K (2006). Inhibition of NF-kappa B activation through targeting I kappa B kinase
by celastrol, a quinone methide triterpenoid. Biochem Pharmacol.

[23] Chen Q, Cederbaum AI (1997). Menadione cytotoxicity to Hep G2 cells and protection by activation of
nuclear factor-kappaB. Mol Pharmacol.

[24] Luo H, Zhang H, Zhang Z, Zhang X, Ning B, Guo J (2009). Down-regulated miR-9 and miR-433 in human gastric
carcinoma. J Exp Clin Cancer Res.

[25] Hildebrandt MA, Gu J, Lin J, Ye Y, Tan W, Tamboli P (2010). Hsa-miR-9 methylation status is associated with cancer development and
metastatic recurrence in patients with clear cell renal cell
carcinoma. Oncogene.

[26] Sun C, Li N, Yang Z, Zhou B, He Y, Weng D (2013). miR-9 regulation of BRCA1 and ovarian cancer sensitivity to cisplatin
and PARP inhibition. J Natl Cancer Inst.

[B27] Huang H, Benzonana LL, Zhao H, Watts HR, Perry NJ, Bevan C (2014). Prostate cancer cell malignancy via modulation of HIF-1alpha pathway
with isoflurane and propofol alone and in combination. Br J Cancer.

[28] Mammoto T, Mukai M, Mammoto A, Yamanaka Y, Hayashi Y, Mashimo T (2002). Intravenous anesthetic, propofol inhibits invasion of cancer
cells. Cancer Lett.

[29] Melamed R, Bar-Yosef S, Shakhar G, Shakhar K, Ben-Eliyahu S (2003). Suppression of natural killer cell activity and promotion of tumor
metastasis by ketamine, thiopental, and halothane, but not by propofol: mediating
mechanisms and prophylactic measures. Anesth Analg.

[B30] Rushworth SA, Murray MY, Barrera LN, Heasman SA, Zaitseva L, MacEwan DJ (2012). Understanding the role of miRNA in regulating NF-kappaB in blood
cancer. Am J Cancer Res.

[31] Wan HY, Guo LM, Liu T, Liu M, Li X, Tang H (2010). Regulation of the transcription factor NF-kappaB1 by microRNA-9 in
human gastric adenocarcinoma. Mol Cancer.

[32] Arora H, Qureshi R, Jin S, Park AK, Park WY (2011). miR-9 and let-7g enhance the sensitivity to ionizing radiation by
suppression of NFkappaB1. Exp Mol Med.

